# Medical expenditure for patients with hemophilia in urban China: data from medical insurance information system from 2013 to 2015

**DOI:** 10.1186/s13023-020-01423-7

**Published:** 2020-06-05

**Authors:** Guang-wen Gong, Ying-chun Chen, Peng-qian Fang, Rui Min

**Affiliations:** 1 School of Medicine and Health Management, Tongji Medical College, Huazhong University of Science and Technology, Wuhan, China; 2 School of Public Health, Tongji Medical College, Huazhong University of Science and Technology, Wuhan, China

**Keywords:** Medical expenditure, Hemophilia, China, Urban employee basic medical insurance, Urban residence basic medical insurance

## Abstract

**Background:**

Hemophilia, a high-cost disease, is the only rare disease covered by basic medical insurance in all province of China. However, very few studies have estimated the medical expenditure of patients with this rare disease Therefore, this study is aimed at evaluating the medical expenditure of patients with hemophilia and identifying its determinants.

**Methods:**

The study population included 450 patients with hemophilia who were extracted from the national insurance database between 2014 and 2016. An independent-sample Kolmogorov–Smirnov test was performed to compare the medical expenditure of patients with hemophilia covered under urban employee basic medical insurance (UEBMI) and urban residence basic medical insurance (URBMI). Quantile regression analysis was conducted to explore the factors that affect the medical expenditure of patients with hemophilia.

**Results:**

The total annual medical expenditure of patients with hemophilia in 2013, 2014, and 2015 had median of ¥7167 (US$ 1156), ¥3522 (US$ 577), and ¥4197 (US$ 677), respectively. The median medical expenditures of patients with hemophilia covered by UEBMI were ¥10,991 (US$ 1773), ¥2301 (US$ 377) and ¥8074 (US$ 1302), those of patients covered by URBMI were ¥4000 (US$ 645), ¥5717 (US$ 937) and ¥3141 (US$ 507) from 2013 to 2015. The differences in the medical expenditure of patients with hemophilia between UEBMI and URBMI from 2013 to 2015 were statistically significant. The number of admissions and the number of hospital days were statistically significant and positive for all quantiles. The types of medical service were statistically significant and negative for 50th quantile, and the reimbursement ratio was statistically significant and positive for 50th and 75th quantiles. (*p* < 0.05).

**Conclusion:**

The medical expenditure of patients with hemophilia was lower than that of patients with other common rare diseases that were not included in the scope of basic medical insurance reimbursement. It was also observed that the medical expenditure was mainly influenced by the severity of disease, and partly affected by the reimbursement rate.

## Background

Hemophilia is a hereditary bleeding disorder that results from the lack or deficiency of clotting factors VIII or IX. The disease is characterized by the spontaneous bleeding of the joints, muscles, viscera and deep tissues and difficulty in stopping the bleeding. It usually requires lifelong treatment and results in a considerable social and economic burden [1, 2].

Although China has established a national hemophilia registration system, the registration rate of patients with hemophilia is extremely low, and the prevalence of hemophilia in mainland China remains unclear [3]. It is estimated that in the worldwide population, approximately 400,000 individuals have hemophilia; and the total number of patients with hemophilia in China is 109,426 [4, 5]. In a meta-analysis involving existing data, the overall weighted prevalence of hemophilia is 3.6 per 100,000 individuals, and the prevalence among males is 5.5 per 100,000 individuals in mainland China [6]. Hemophilia is a severe health issue in China because it is associated with high treatment cost. According to a research on the treatment cost of hemophilia in Guangdong Province, 12.1% of treatment cost was more than ¥240,000 (US$ 34,782) in 2008, 6.1% treatment cost was between ¥120,000 (US$ 17,391) and ¥240,000, 15.2% of treatment cost was between ¥60,000 (US$ 8695) and ¥120,000, and 66.6% of treatment cost was less than ¥60,000 in 2008.The quality of life of children with hemophilia is significantly lower than that of healthy children; moreover, their treatment cost is nearly five times higher than that of a healthy family, more than half of families with hemophilia cannot completely afford the therapy in China [7–9]. In 2012, the Chinese Ministry of Health announced that hemophilia is a high-cost disease that should be prioritized to ease their economic burden. Health insurance is an effective way to share the economic risk of diseases. The Chinese government has established a basic medical insurance system and currently it. The current basic medical insurance in China comprises of three main insurance schemes: urban employee basic medical insurance (UEBMI), new rural cooperative medical system (NRCMS), and urban residence basic medical insurance (URBMI). The UEBMI, which was initiated in 1998, covers urban employees and retired employees; the URBMI, established in 2003, covers urban residents, including children, students, elderly people without previous employment, and unemployed people; and the NRCMS, launched in 2007, covers rural residents. The coverage by basic medical insurance is nearly universal, that is, it has exceeded 95% of the population since 2011 [10]. The financing source of UEBMI comes from employers (6% of total wages) and employees (2% of their wages). The UEBMI and the NRCMS are financed mainly by the government, with minimal individual premium contributions. The UEBMI and URBMI are administered by the Ministry of Human Resources and Social Security, whereas the NRCMS is administered by the Health and Family Planning Commission and run all three are by the local government. According to the local development, each local government sets the local minimum deduction of three insurance schemes, reimbursement cap and an individual’s share of medical costs, with the principle of keeping expenditure within income. This is critical as the differences in economic development and health insurance design can result in the disparity of insurance benefit packages [11–16]. ^−^

The Chinese government has also designed the National Reimbursement Drug List (NRDL), which is divided into class A and class B. All drugs in NRDL were purchased by public bidding. Class A drug were 100% reimbursed by all the three insurance schemes, but class B were partially reimbursed by three insurance schemes (10–90%) [17]. Drugs for hemophilia are included in the NRDL, which were reimbursed by the basic medical insurance of each province. According to the National Reimbursement Drug Lis (2004 edition), the freeze-dried human coagulation factor VIII was identified as a class A drug [18]. In the 2009 edition, gene recombinant coagulation factor VIII was identified as a class B drug [19]. In the 2017 edition, human clotting factor VIII was identified as a class A drug, whereas human recombinant coagulation factor IX, a class B drug [20, 21]. Drugs for hemophilia are included in the NRDL, which were reimbursed by the basic medical insurance of each province. Thus, hemophilia is the only rare disease that is covered by basic medical insurance in each province in China.

Basic medical insurance focuses on the diseases with a large number of patients, and provides little coverage of rare diseases. This may lead to patients with rare diseases experience high financial burden for patients with rare diseases. Although hemophilia is the only rare disease covered by basic medical insurance, the disparities in insurance benefit packages of basic medical insurance in each province lead to the disparities of medical expenditure for patients with hemophilia. Few studies have explored the disparities in the insurance benefit packages of basic medical insurance affecting the medical expenditure of patients in China. This study aimed to evaluate the medical expenditure of patients with hemophilia, and identify its determinants in mainland China.

## Methods

### Data source

The data used in this study were derived from the national insurance database which is operated by China Healthcare Insurance Research Association (CHIRA). CHIRA extracted data from the medical insurance management system covering the whole country by retrospective sampling method. Sample cities were selected by stratified sampling, the sample size is 5% of the total number of participants of sample cities, and the case was selected by sampling space. The data from 2014 to2016 contain information about disease diagnosis and medical expenditure of patients covered by UEBMI and URBMI from 2013 to 2015. All patients diagnosed with hemophilia and recorded in the database were selected (155, 148, and 147 patients with hemophilia in 2013, 2014, and 2015,, respectively).

### Indicators definitions

For the purpose of this study, medical expenditure was defined as the total direct costs of seeking healthcare services for a patient with hemophilia, which was the sum of treatment cost of each visit in a year. Indirect costs, such as the costs of transportation and special diets and wages lost due to illness, were not considered in this study. Furthermore, medical expenditure was categorized into two components—expenses within and beyond the reimbursement coverage of basic health insurance in China. Expenses beyond the reimbursement coverage of basic health insurance paid by a person is referred to as the out-of-pocket costs. The proportion of medical expenses within the reimbursement coverage of basic health insurance accounted for the medical expenditure is defined as the reimbursement rate (RR) of basic medical insurance. The insurance benefit packages were represented by RR in this study:

$$ \mathrm{RR}=\mathrm{total}\ \mathrm{expenditure}\ \mathrm{reimbursed}\ \mathrm{by}\ \mathrm{basic}\ \mathrm{medical}\ \mathrm{insurance}/\mathrm{total}\ \mathrm{medical}\ \mathrm{expenditure}\times 100\% $$

### Statistical analysis

Statistical analysis was performed using SPSS 24.0 for Windows (IBM Corp., Armonk, NY, USA) and STATA/SE 15. Descriptive analysis and independent-sample Kolmogorov–Smirnov test were performed by SPSS 24.0. The median and interquartile ranges of the medical expenditures of patients with hemophilia were calculated via descriptive analysis. An independent-sample Kolmogorov–Smirnov test was carried out to compare medical expenditure of patients with hemophilia covered by the two types of health insurance schemes. Quantile regression analysis was performed with STATA/SE 15 to explore the factors that affect medical expenditure of patients with hemophilia. Statistical significance was considered at *p* < 0.05.

## Results

### Descriptive analysis

Table 1 presents the medical information of patients with hemophilia. The treatment costs of 450 patients with hemophilia from 2013 to 2015 were obtained. Patients with hemophilia were mainly male, which accounted for 89.33% of the participants. Among the total participants, 52.10% were covered by UEBMI and, 47.90% were covered by URBMI. During the study year, 50% of the patients used the out-patient service, 42.67% used the in-patient service only, and 7.33% used both out-patient and in-patient services. It was also found that 22% RR was less than 30, 23.33% RR was between 30 and 60%, 47.56% RR was between 60 and 90%, and 17.11% RR was higher than 90%.

**Table 1:** Medical expenses and reimburse ration distribution of patients with haemophilia

	Indicator	Median	IQR	Non-parametric Test
outpatient	medicalexpensest			*P* = 0.000
	UEBMI	799.56	813.60	
	URBMI	43.48	552.22	
	reimburse ratio,%			*P* = 0.002
	UEBMI	80.00	100.00	
	URBMI	70.00	57.13	
inpatient	medicalexpensest			*P* = 0.000
	UEBMI	5995.80	5689.68	
	URBMI	3194.93	4832.23	
	reimburse ratio,%			*P* = 0.000
	UEBMI	85.67	19.20	
	URBMI	63.07	30.02	

Table 2 presents the average annual medical expenditure of patients with hemophilia. The total annual medical expenditures of patients with hemophilia in 2013, 2014, and 2015 had median of ¥7167 (US$ 1156), ¥3522 (US$ 577), and ¥4197 (US$ 677), respectively. The medical expenditures of patients with hemophilia covered by UEBMI had median of ¥10,991 (US$ 1773), ¥2301 (US$ 377) and ¥8074 (US$ 1302). By comparison, the medical expenditures of patients with hemophilia ¥4000 (US$ 645), ¥5717 (US$ 937) and ¥3141 (US$ 507) from 2013 to 2015. The differences in medical expenditure of the patients with hemophilia between UEBMI and URBMI from 2013 to 2015 were statistically significant.

**Table 2:** The generalised estimating equation analysis of medical expenses

Parameter	B	Std.Error	95% wald confidence Internal		hypothesis test		
			Lower	Upper	wald chi-square	df	*p*-value
intercept	5169.088	2572.454	127.170	10,211.006	4.038	1	0.044
[region = 1]	1289.574	979.440	− 630.093	3209.241	1.734	1	0.188
[region = 2]	1700.035	1349.644	− 945.219	4345.288	1.587	1	0.208
[region = 3]	0b						
[gender = 1]	− 544.542	1937.713	− 4342.389	3253.305	0.079	1	0.779
[gender = 2]	0b						
age	−400.917651	707.808	− 1788.195	986.360	0.321	1	0.571
[types of BMI = 1]	360.977	1220.132	− 2030.437	2752.391	0.088	1	0.767
[types of BMII = 2]	0b						
[grades of medical institution = 0]	159.418	759.276	− 1328.734	1647.571	0.044	1	0.834
[grades of medica linstitution = 1]	9223.120	#######	−13,906.490	32,352.730	0.611	1	0.434
[grades of medica linstitution = 2]	1986.024	1177.259	−321.361	4293.410	2.846	1	0.092
[grades of medical institution = 3]	0b						
[types of medical service = 1]	− 5085.887	772.286	− 6599.540	− 3572.233	43.369	1	0.000
[types of medical service = 2]	0b						
reimbursement ratio	27.518	10.756	6.437	48.600	6.546	1	0.011
scale	69,869,737.205						

### Quantile regression

Table 3 presents quantile regression of medical expenditure. It was noted that age of the participants was not statistically significant for all quantiles. The types of medical service were statistically significant and positive for 50th and 95th quantiles. RR was statistically significant and positive for 50th and 75th quantiles. The number of admissions and the number of hospital days were statistic significant and positive for all quantiles (Table 3, *p* < 0.05).

**Table 3:** The generalised estimating equation analysis of reimbursement ratio

Parameter	B	Std.Error	95% wald confidence Internal		hypothesis test		
			Lower	Upper	wald chi-square	df	*p*-value
intercept	11.876	8.858	−5.486	29.238	1.797	1	0.180
[region = 1]	21.074	3.556	14.104	28.043	35.124	1	0.000
[region = 2]	10.397	3.511	3.516	17.278	8.770	1	0.003
[region = 3]	0a						
[gender = 1]	38.345	7.054	24.519	52.170	29.549	1	0.000
[gender = 2]	0a						
age	−2.587	2.046	−6.598	1.424	1.598	1	0.206
[types of BMI = 1]	15.129	3.252	8.756	21.503	21.649	1	0.000
[types of BMII = 2]	0a						
[grades of medical institution = 0]	7.901	3.409	1.219	14.583	5.371	1	0.020
[grades of medica linstitution = 1]	15.348	3.952	7.602	23.093	15.083	1	0.000
[grades of medica linstitution = 2]	−0.060	3.004	−5.948	5.828	0.000	1	0.984
[grades of medical institution = 3]	0a						
[types of medical service = 1]	−3.431	2.951	−9.214	2.353	1.352	1	0.245
[types of medical service = 2]	0a						
medical expenses	0.000	0.000	0.000	0.001	2.844	1	0.092
scale	687.056						

## Discussion

Medical expenditure, which is not only relate related to the severity of diseases but also affected by technological progress, variation in medical practice, and health system characteristics, reflect the consumption of health resources and services by patients [22–24]. A previous study revealed that the median total treatment costs of patients with 8 certain rare diseases in 2013, 2014 and 2015 were ¥6548, ¥3752, ¥5378, respectively [25]. In our study, the median of the annual health expenditure of patients with hemophilia in 2014 was ¥7167, which was significantly higher than that in the previous study. The annual health expenditure for patients with hemophilia in 2014 to 2015 had median of ¥ 3522 and ¥4197, which were lower than the previous study. These values differed probably because the drugs used to treat hemophilia were listed in basic medical insurance reimbursement schemes. These drugs purchased using basic medical insurance funds are procured through bidding and negotiation, and it can significantly reduce drugs’ prices [26].

In this study, it was observed that the more frequent hospital admission and the longer the hospital stays, the higher the medical expenditure of patients with hemophilia. This also meant these that patients suffered from severe hemophilia and endured high annual treatment cost, which was consistent with the previous results [27–29].

RR was significant positive in 50th and 70th quantiles possibly because of the difference in health service utilization among different insurance schemes. Previous studies indicated that patients covered by different health schemes have different medical expenses in China. Patients covered by UEBMI with a high RR use more health services, which entail additional treatment costs. The cost of hospitalization, length of stay and frequency of hospitalization of patients in rural areas were lower than those of patients in urban areas [30, 31].

The types of medical service were significant negative in the 50th quantile. The common treatment of patients with hemophilia was coagulation factor injection, which was mainly provided by the outpatient department. Patients that repeatedly made use of the outpatient services in a year also had a considerable amount of medical cost [32]. In our study, the annual treatment cost was not statistically different between adults and children. This result was consistent with the hemophilia-associated cost in Portugal [33].

The limitations of this study should also be noted. Firstly, patients with hemophilia covered by the NRCMS were excluded because our data did not provide relevant information. Secondly, this study did not pay attention to the socio-economic factors, health needs and service utilization of patients with hemophilia because relevant data were not available.

## Conclusion

Hemophilia, which is a costly but treatable rare disease, is included in the scope of basic medical insurance reimbursement in China. The medical expenditure of patients with hemophilia was lower than that of other common rare diseases that are not included in the scope of basic medical insurance reimbursement. The medical expenditure of patients with hemophilia was mainly influenced by disease severity, and partly affected by the RR. Further studies are required to focus on the effects of basic medical insurance on the medical expenditures of patients with hemophilia, design a reasonable reimbursement policy that may reduce treatment costs and improve the efficiency of health services.

## Data Availability

The data used in this study were obtained with permission from the China Healthcare Insurance Research Association, information related to patients’ privacy was not extracted. The corresponding author has full access to all the data used in the study.

## Abbreviations

UEBMI: Urban employee basic medical insurance
NRCMS: New rural cooperative medical system
URBMI: Urban residence basic medical insurance
