# Correction to: Medical expenditure for patients with hemophilia in urban China: data from medical insurance information system from 2013 to 2015

**DOI:** 10.1186/s13023-020-01524-3

**Published:** 2020-09-08

**Authors:** Guang-wen Gong, Ying-chun Chen, Peng-qian Fang, Rui Min

**Affiliations:** 1 School of Medicine and Health Management, Tongji Medical College, Huazhong University of Science and Technology, Wuhan, China; 2 School of Public Health, Tongji Medical College, Huazhong University of Science and Technology, Wuhan, China

**Correction to: Orphanet J Rare Dis 15, 137 (2020)**

**https://doi.org/10.1186/s13023-020-01423-7**

Following the publication of the original article [1], the authors unfortunately became aware of errors in Tables 1, 2 and 3.

**Table 1:** Descriptive analysis of medical information of patients with hemophilia

Items (n)	NO. of patients	Proportion(%)
**Year**		
2013	155	34.44
2014	148	32.89
2015	147	32.67
**Regions**		
East region	270	60.00
Central region	87	19.33
West region	93	20.77
**Gender**		
Male	1386	89.33
Female	97	10.77
**Age**		
0–18	130	28.89
> 18	320	71.11
**Types of basic medical insurance**		
Urban employee basic medical insurance	234	52.00
Urban resident basic medical insurance	216	48.00
**Types of medical services**		
Outpatient	225	50.00
Inpatient	192	42.67
Outpatient & Inpatient	33	7.33
**Total reimbursement ratio**		
Less than 30%	54	12
30 to 60%	105	23.33
60 to 90%	214	47.56
More than 90%	77	17.11

**Table 2:** the average annual medical expenditure of patients with hemophilia

		Median (¥. RMB)	IQR(¥. RMB)	Non-parametric Test
2013	Total	7167	17809	
	UEBMI	10991	18533	0.002
	URBMI	4000	9211	
2014	Total	3522	14733	
	UEBMI	2301	11533	0.031
	URBMI	5717	25282	
2015	Total	4197	15659	
	UEBMI	8074	19826	0.037
	URBMI	3141	13734

**Table 3:** Quantile regression of medical expenditure for outpatient with heamophilia

Covariates	0.05	0.25	0.5	0.75	0.95
Age	17.14478	252.6427	− 341.8183	843.4473	4008.514
Types of medical service	− 353.581	− 778.177	− 2042.498 *	− 455.260	########
Number of admissions	1142.936 ***	2474.729***	3523.898 ***	3691.233 ***	17,886.04 ***
Number of hospital days	94.37434 ***	204.157***	36.439***	851.016 ***	2465.452 **
reimbursement rate	0.119	9.132	36.439*	157.886**	331.895

* *p* < 0.05, ** *P* < 0.01, *** *p* < 0.001

The correct Tables are provided here below:
